# Transumbilical Laparoscopic-Assisted (TULA) Surgery for Treating Abdominal Pathologies in Newborns: A Retrospective Single-Center Experience

**DOI:** 10.3390/children13030338

**Published:** 2026-02-27

**Authors:** Giada Loria, Roberta Aurora Aversa, Alessandra Fichera, Agnese Bartolone, Vincenzo Di Benedetto, Maria Grazia Scuderi

**Affiliations:** Pediatric Surgery Unit, Department of Advanced Medical and Surgical Sciences G. Ingrassia, Policlinico G. Rodolico—San Marco Hospital, University of Catania, 95124 Catania, Italy

**Keywords:** neonatal surgery, minimally invasive surgery, transumbilical, TULA, laparoscopic-assisted surgery, pediatric surgery

## Abstract

**Background:** Transumbilical laparoscopic-assisted (TULA) surgery is a minimally invasive technique that combines laparoscopic exploration with extracorporeal surgical management, offering potential advantages in neonatal abdominal surgery. However, comparative data with conventional open surgery in neonates remain limited. This study reports our single-center experience with TULA and compares its outcomes with those of a matched cohort of neonates undergoing open surgery. **Methods:** We performed a retrospective study on neonatal patients (<28 days of life) treated at our Pediatric Surgery Unit between 2015 and 2023. Twenty-five neonates underwent TULA for various intra-abdominal malformations. Each TULA patient was matched in a 1:2 ratio with neonates treated with open surgery based on gestational age, birth weight, and underlying diagnosis, resulting in a matched cohort of 50 patients. Primary outcomes included operative and anesthesia times, conversion rate, postoperative complications, length of hospital stay, and mortality. **Results:** The TULA cohort included 11 males and 14 females, with a mean gestational age of 37.5 ± 1.9 weeks and a mean birth weight of 2989 ± 675 g. Indications comprised intestinal malrotation, ileal atresia, duodenal stenosis, meconium ileus, and other abdominal pathologies. Mean operative time was comparable between groups (116 ± 37 min in the TULA group vs. 137 ± 65.9 min in the open surgery group; *p* = 0.52). Conversion from TULA to open surgery occurred in 16% of cases. No significant differences were observed in major postoperative complications or length of hospital stay between groups (*p* > 0.05). No mortality was reported. **Conclusions:** TULA represents a safe and effective surgical option for selected neonatal abdominal pathologies, with outcomes comparable to conventional open surgery. When performed in specialized centers with appropriate patient selection and multidisciplinary expertise, TULA offers favorable safety and cosmetic results.

## 1. Introduction

In pediatric surgery, one of the main objectives is to achieve desirable outcomes while minimizing surgical trauma and improving aesthetic results. Neonatal surgical diseases represent a critical component of pediatric healthcare, demanding highly specialized centers and multidisciplinary teams. Congenital anomalies and acquired abdominal pathologies in newborns pose significant challenges, contributing heavily to neonatal morbidity and mortality, and placing substantial economic and structural demands on healthcare systems. Conventionally, “standard” neonatal surgery has relied on open laparotomy. This traditional approach, while providing excellent visibility and direct, rapid access to abdominal organs, involves significant surgical trauma, considerable postoperative pain, prolonged recovery times, and an increased risk of wound complications. The advent of neonatal laparoscopy began in the late 20th century and was initially met with skepticism due to concerns regarding the size and fragility of neonates. However, advancements in technology and surgical techniques have led to increased acceptance and application in pediatric surgery [1,2,3].

Over time, driven by technological advances and expertise spreading, neonatal laparoscopy has become a preferred approach for treating congenital pathological conditions, with the aim of offering better postoperative outcomes, minimized scarring, and faster recovery times [4,5,6]. Despite these advantages, challenges remain, including the requirement for specialized training, the possibility of injuring adjacent organs, and longer surgical durations when performed by less experienced practitioners [7,8]. For this reason, open surgery remains the mainstay treatment across several centers, particularly for neonatal surgery [9].

The transumbilical laparoscopic-assisted (TULA) procedure combines the advantages of both laparoscopic treatment and open surgery: minimal aesthetic scars, surgical performance comparable to traditional surgery, quick postoperative recovery, and satisfactory outcomes [10]. TULA is performed through a single incision at the umbilical scar, where a trocar is placed, and pneumoperitoneum is established. The target anatomy is then exteriorized through the umbilicus, allowing for manual surgical treatment [11]. Historically, the single-incision umbilical approach was first introduced by Tan and Bianchi in 1986 for pyloromyotomy [12].

The use of minimally invasive techniques in newborns was initially limited by the extremely confined working space, instrument size, and the unique physiology of neonates, which makes them more sensitive to the adverse effects of peritoneal carbon dioxide insufflation [13,14]. With the development of high-quality instruments and increasing experience with CO_2_ pressure adaptation, broad clinical experience has been gained across neonatal surgery [14,15].

We present our experience with the TULA surgical technique for intra-abdominal malformations in newborns, comparing outcomes with a matched cohort of neonates treated with traditional open surgery.

## 2. Materials and Methods

The study was conducted according to the guidelines of the Declaration of Helsinki. Ethical review and approval were waived due to the retrospective nature of the study and the use of anonymized clinical data collected as part of routine care.

We conducted a retrospective study on neonatal patients treated at the Pediatric Surgery Unit of G. Rodolico–S. Marco Hospital in Catania (Italy) between 2015 and 2023. Clinical and demographic data analyzed included prenatal diagnosis, gestational age, birth weight, age at surgery, cause of intestinal obstruction, surgical and anesthesia times, complications, and follow-up.

Twenty-five patients were enrolled based on the following inclusion criteria:

- Neonatal age (<28 days of life);
- Use of a TULA approach.

A retrospective matching method was employed, selecting control patients based on three criteria: gestational age, birth weight, and identical underlying primary diagnosis, resulting in a matched cohort of 50 patients.

**Figure 1:**
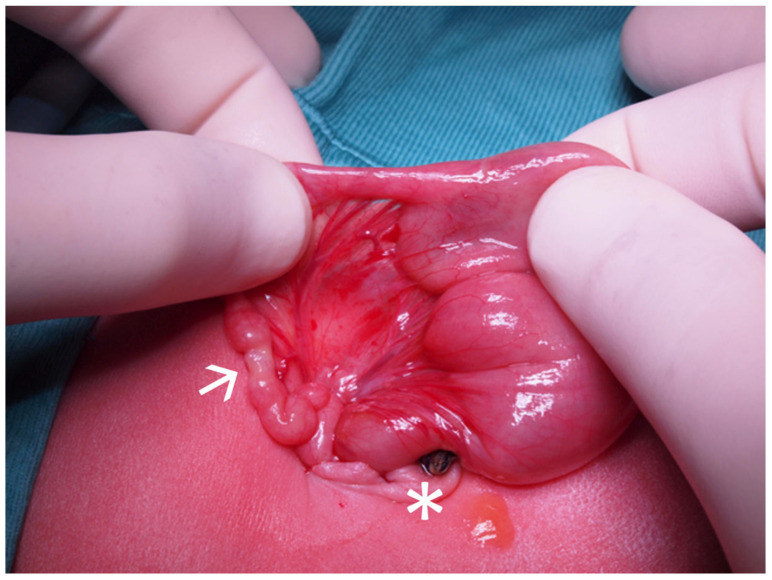
Exteriorization of atretic intestinal segment through the TULA technique. The asterisk (*) indicates the umbilicus, and the arrow (→) indicates the atretic segment of the intestine.

Eligibility for TULA was discussed by a multidisciplinary team including surgeons, neonatologists, and anesthesiologists. All patients underwent general anesthesia with orotracheal intubation and local anesthetic infiltration. All procedures were performed by a single senior pediatric surgeon with extensive advanced training in minimally invasive surgery. The potential learning curve associated with this hybrid method was substantially mitigated by the surgeon’s prior extensive experience in both standard neonatal laparoscopy and conventional open surgery. For the TULA procedure, access to the abdomen was achieved exclusively through the natural umbilical scar. Each procedure began with a semicircular supraumbilical incision; a 10 mm trocar was placed using the open Hasson technique to enter the peritoneal cavity. Following this, intra-abdominal pressure was maintained at 5–8 mmHg through CO_2_ insufflation at 0.5–1 L/min to establish pneumoperitoneum. A 10 mm 0° laparoscope with 5 mm operative channel was used for abdominal exploration and target anatomy identification. Adhesiolysis was performed when required. Once the pathology was identified, the target organ was exteriorized through the umbilical incision (Figure 1) and with the help of a Joanne laparoscopic forceps. The procedure was then completed extracorporeally to ensure optimal ergonomics.

**Figure 2:**
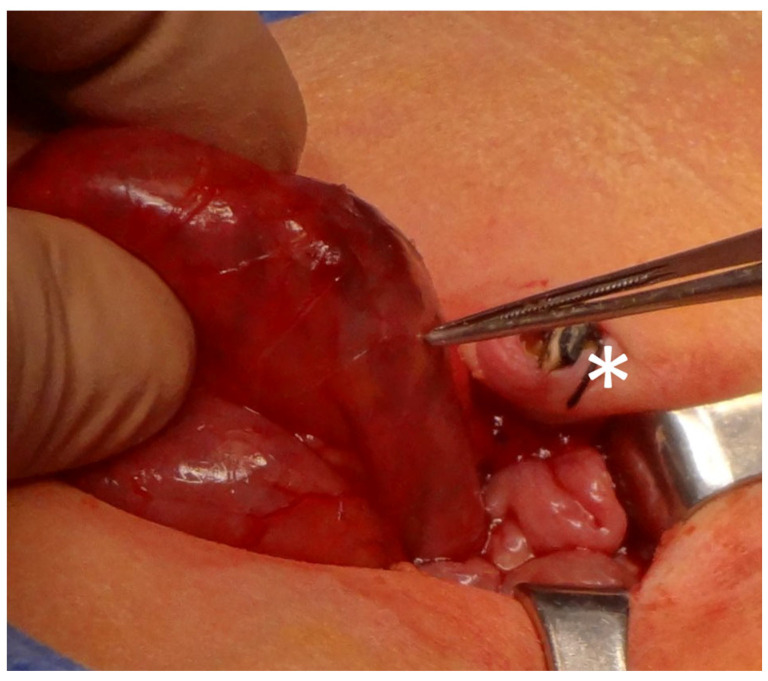
Longitudinal supra-umbilical incision for open surgery technique. The umbilical scar is pointed by the asterisk (*).

In the matched open surgery cohort, the abdominal cavity was accessed via a laparotomy chosen according to the pathology. Median longitudinal incision or supraumbilical transverse incision for intestinal pathologies (Figure 2) and Pfannenstiel incision for ovarian lesions are the access of choice.

Conversion to open surgery was performed when TULA was not feasible due to ventilation issues, extreme intestinal dilation, or dissection problems, comprising xifo-umbilical laparotomy or lateral umbilical incision extension.

The surgical technique selected for each pathology was the same in both the TULA and open-surgery cohorts. No mechanical staplers were used.

In cases of intestinal malrotation, adhesiolysis and the Ladd procedure were performed. Surgical management of mesenteric lymphatic malformations consisted of complete excision.

In cases of atresia and intestinal duplication, as well as in patients with Meckel’s diverticulum, the procedure consisted of bowel resection followed by termino-terminal anastomosis using hand-sewn techniques with absorbable sutures. When bowel perforation was identified, the same approach was adopted for meconium ileus, instead of performing an enterotomy followed by primary closure.

For ovarian cysts, a parenchyma-preserving stripping technique was performed.

Complications were evaluated according to Clavien-Madadi Classification (CMC) [16], distinguishing the major ones as every event matching the grading 3B or greater. Primary and secondary outcomes included mortality, total hospitalization time, postoperative complications, and time to bowel motility recovery.

Statistical analysis used Student’s *t*-test for continuous variables and Chi-square or Fisher’s exact test for categorical variables. A *p*-value < 0.05 was considered statistically significant.

## 3. Results

**Figure 3:**
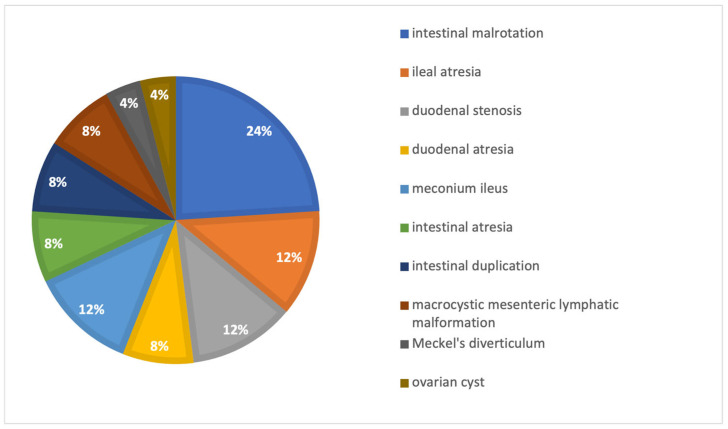
Primary diagnosis in the TULA surgery cohort.

Between 2015 and 2023, 264 newborns underwent abdominal surgery at our institution. Twenty-five (9.5%) were treated using TULA, consisting of 11 males (44%) and 14 females (56%). Mean gestational age was 37.5 ± 1.9 weeks, with mean birth weight 2989 ± 675 g (range 1925–4000). Conditions treated are displayed in Figure 3 and included intestinal malrotation (24%), ileal atresia (12%), duodenal stenosis (12%), duodenal atresia (8%), meconium ileus (12%), multiple intestinal atresia (8%), intestinal duplication (8%), macrocystic mesenteric lymphatic malformation (8%), Meckel’s diverticulum (4%), and ovarian cyst (4%). Prenatal diagnosis was made in six cases (24%): three intestinal atresias, and one each of duodenal stenosis, mesenteric lymphangioma, and meconium ileus. Associated pathologies included Down syndrome (15%), patent foramen ovale (15%), atrial septal defect (3%), patent ductus arteriosus (3%), sepsis (11%), and respiratory distress (4%). Mean age at surgery was 5.3 ± 5 days (median 4, range 1–20).

Mean anesthesia time was 188 ± 36.7 min (median 180), and mean operative time was 116 ± 37 min (median 100). Conversion rate was 16%. Mean hospital stay was 30.2 ± 16 days (median 26, range 15–60). Major complications occurred in one case (4%) involving a preterm neonate with complex duodeno-ileal atresia and malrotation who developed postoperative adhesive bowel obstruction, successfully managed with an open re-laparotomy. Mean follow-up was 68 months, with no mortality.

**Figure 4:**
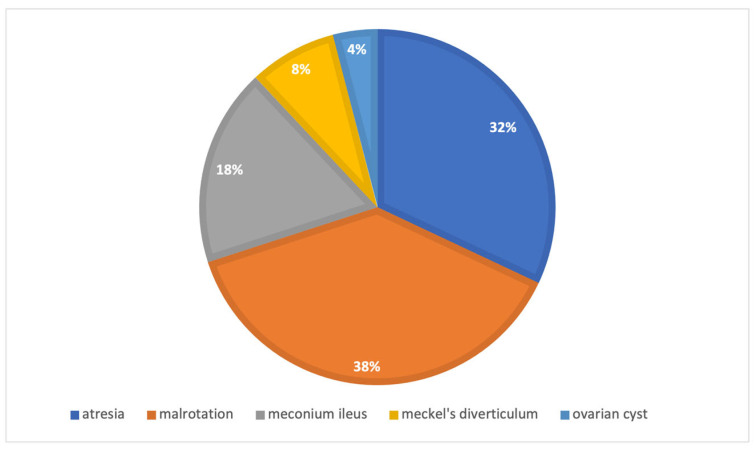
Primary diagnosis in the open-surgery cohort.

The matched open surgery cohort comprised 50 newborns with a 1:1.3 male-to-female ratio. Baseline demographics and clinical characteristics for both groups are summarized in Table 1, demonstrating no statistically significant differences and confirming the adequacy of the matching process. Mean birth weight was 2709 ± 535 g (*p* = 0.19), with mean gestational age 34.2 ± 1.9 weeks (*p* = 0.10). As illustrated in Figure 4, primary diagnoses were atresias (32%), malrotation (38%), meconium ileus (18%), Meckel’s diverticulum (8%), and ovarian cyst (4%). Prenatal diagnosis was present in 18% (*p* = 0.55). Thirty percent had associated cardiac anomalies. Mean age at surgery was 9.2 ± 9.2 days (median 4.5, range 1–28) (*p* = 0.24).

A comparative summary of surgical outcomes is provided in Table 2. Mean operative time was 137 ± 65.9 min (median 130) (*p* = 0.52), and mean anesthesia time was 203 ± 91.2 min (median 180) (*p* = 0.74). Mean hospital stay was 38.7 ± 13 days (*p* = 0.14). Major complications occurred in 3 cases (6%), which were two cases of adhesive bowel obstruction and one anastomotic leak, requiring second surgical intervention (*p* = 0.72). No mortality was recorded, with a mean follow-up of 73 months.

## 4. Discussion

Neonatal laparoscopic surgery has advanced significantly, offering reduced postoperative pain, shorter recovery times, and smaller incisions compared to open surgery [1,2,3]. However, limited working space complicates instrument maneuverability and increases injury risk, requiring specialized skills. The steep learning curve, variable equipment availability, and institutional experience affect procedural outcomes. Despite these challenges, technological advancements continue to enhance safety and efficacy [1,2,3].

Intestinal obstruction in newborns is rare but severe, with a risk of rapid deterioration. Common causes within 28 days include atresias, meconium ileus, Hirschsprung disease, malrotation, necrotizing enterocolitis, and incarcerated inguinal hernia [17,18,19,20,21,22,23].

Ovarian pathologies, including cysts and tumors, also represent conditions amenable to minimally invasive surgery [24,25].

The feasibility of minimally invasive surgery (MIS) in newborns has been extensively documented, documenting low conversion rates, acceptable complication rates, and no mortality if performed by experienced hands. Furthermore, neonatal MIS is associated with shorter hospital stays, reduced postoperative pain, faster re-alimentation, and cosmetic results compared to open surgery [26,27].

TULA emerged in the early 2000s as a minimally invasive approach utilizing the natural umbilical scar, avoiding additional incisions [10,11]. TULA demonstrated comparable safety profiles to traditional laparoscopy for both intestinal and urologic surgery [28,29].

TULA utilizes neonatal organ mobility to perform surgery outside the abdominal cavity, reducing CO_2_ insufflation effects and restricted space disadvantages, with the operative scope exploration enabling the identification of associated anomalies beyond the primary target [30]. In experienced hands, TULA combines laparoscopic and open technique benefits, providing adequate exposure with excellent cosmetic results [31,32].

Our study reports our TULA experience for neonatal intestinal obstruction and abdominal pathologies. Of 264 neonates, 25 (9.5%) received TULA surgery. To the best of our knowledge, this is among the few studies comparing TULA with matched open surgery controls.

A multidisciplinary candidate selection was accomplished, evaluating CO_2_ insufflation tolerance and technical feasibility. Both groups showed comparable baseline characteristics. Birth weight was similar, though gestational age differed, with more premature infants in the open surgery group, possibly reflecting the cautious MIS selection performed by our team. Age at surgery was comparable.

Pathologies treated were consistent with the literature, predominantly intestinal malrotation, atresias, and meconium ileus (12%). The matched cohort had a similar diagnostic distribution. Prenatal diagnosis rates were low in both groups (24% vs. 18%), highlighting the need for improved screening and timely referral.

### 4.1. Surgical Outcomes

Even though statistical significance was not reached, the mean operative time was shorter in the TULA group. This finding contrasts with reports describing longer operative times for minimally invasive surgery [33], but it may be explained by the careful selection of clinically stable patients for the MIS procedure.

Mean anesthesia time was longer in the TULA group. This difference may witness the additional time required for patient positioning, establishment of pneumoperitoneum, and laparoscopic exploration inherent to minimally invasive approaches.

The observed conversion rate of 16% falls within the ranges reported in the literature for TULA [31,34]; however, a specific comparison in newborns could not be performed due to the lack of published data. While minimally invasive surgery in neonates is well-documented, comparative studies specifically evaluating TULA versus conventional open surgery remain scarce in the literature. Previous studies in pediatric populations, such as those by Bawazir et al. [11] and Ohno et al. [34], have highlighted that transumbilical approaches yield comparable operative times and significantly better cosmetic outcomes compared to standard multi-port laparoscopy or traditional open surgery. Our comparative analysis aligns with these findings, demonstrating no statistically significant difference in mean operative time between TULA and open surgery (116 vs. 137 min, *p* = 0.52). Furthermore, similar to Gil et al. [32], who evaluated perioperative outcomes in minimally invasive versus open surgery in infants, our data confirms that TULA does not increase the risk of major complications (4% in TULA vs. 6% in open surgery). This direct comparison underlines that, when performed in appropriately selected neonates, TULA successfully bridges the gap between the excellent organ exposure of open surgery and the reduced trauma of laparoscopy, validating its role as a safe alternative to standard laparotomy.

Conversions were necessitated by ventilation instability, marked intestinal dilatation, or technical difficulties during dissection and were managed by extending the umbilical incision in a manner comparable to primary open surgery.

Major postoperative complications (Clavien-Madadi grade ≥ 3B) occurred in one patient (4%) in the TULA group and in three patients (6%) in the open surgery group (*p* = 0.72), in agreement with previously published data [35].

The complication observed in the TULA cohort involved a preterm neonate with complex duodeno-ileal atresia associated with intestinal malrotation, who developed postoperative adhesive bowel obstruction requiring re-laparotomy. This case underscores that conversion does not eliminate the risk of postoperative complications and that complex congenital anomalies remain challenging irrespective of the surgical approach. The comparable complication rates between groups further suggest that TULA does not increase surgical risk in appropriately selected patients.

The total length of hospital stay was shorter in the TULA group, although this difference did not reach statistical significance. While this trend may reflect the potential benefits of reduced surgical trauma and bowel manipulation associated with the TULA approach, the higher degree of prematurity observed in the open surgery cohort should be taken into account when interpreting hospitalization duration.

The excellent cosmetic outcome of TULA, with scars concealed within the umbilical fold, represents an important consideration for families and has been consistently emphasized in the literature [11,26,27,30,31,36], contributing to improved quality of life and parental satisfaction.

No mortality was observed in either group during a mean follow-up of 68 months for the TULA cohort and 73 months for the open surgery cohort, confirming excellent long-term survival with both surgical approaches.

### 4.2. Limitations

This retrospective, single-center study with a relatively small sample size and a single surgeon limits generalizability and external validity. The matched design cannot fully account for unmeasured variables influencing decision-making. We did not systematically assess postoperative pain, analgesic requirements, time to full enteral feeding, or long-term cosmetic satisfaction.

## 5. Conclusions

To our knowledge, this represents one of the largest cohorts of newborns undergoing laparoscopic-assisted minimally invasive surgery for intra-abdominal neonatal malformations. Despite a limited cohort size requiring further evidence, our experience demonstrates that TULA is safe and effective for selected neonatal abdominal pathologies. With appropriate selection, multidisciplinary evaluation, and surgical expertise, TULA offers comparable or superior outcomes to traditional open surgery with excellent cosmetic results. The low conversion rate (16%) and favorable safety profile (4% major complications) support continued use and refinement in specialized pediatric surgery centers. Further prospective, multicenter studies with larger samples are needed to confirm findings and better define optimal TULA indications in neonatal surgery.

## Figures and Tables

**Table 1:** Patient demographics and baseline characteristics.

Characteristic	TULA Group (*n* = 25)	Open Surgery Group (*n* = 50)	*p*-Value
Sex (Male/Female)	11/14	22/28	
Gestational Age (weeks)	37.5 ± 1.9	34.2 ± 1.9	0.10
Birth Weight (grams)	2989 ± 675	2709 ± 535	0.19
Prenatal Diagnosis	6 (24%)	9 (18%)	
Age at Surgery (days)	5.3 ± 5.0 (median 4)	9.2 ± 9.2 (median 4.5)	0.24

**Table 2:** Surgical outcomes and postoperative complications.

Outcome	TULA Group (n = 25)	Open Surgery Group (n = 50)	*p*-Value
Operative Time (min)	116 ± 37.0 (median 100)	137 ± 65.9 (median 130)	0.52
Anesthesia Time (min)	188 ± 36.7 (median 180)	203 ± 91.2 (median 180)	0.74
Major Complications (≥3B)	1 (4%)	3 (6%)	0.72
Length of Stay (days)	30.2 ± 16.0	38.7 ± 13.0	0.14
Conversion Rate	4 (16%)	N/A	-
Mortality	0	0	-

## Data Availability

The data presented in this study are available from the corresponding author upon reasonable request. The data are not publicly available due to privacy reasons.

## Abbreviations

The following abbreviations are used in this manuscript:

CMC	Clavien–Madadi Classification
CO_2_	Carbon Dioxide
MIS	Minimally Invasive Surgery
TULA	Transumbilical Laparoscopic-Assisted
